# Mechanism of IL-12 mediated alterations in tumour blood vessel morphology: analysis using whole-tissue mounts

**DOI:** 10.1038/sj.bjc.6600907

**Published:** 2003-04-29

**Authors:** S A Gerber, J P Moran, J G Frelinger, J A Frelinger, B M Fenton, E M Lord

**Affiliations:** 1 Department of Microbiology & Immunology, University of Rochester Medical Center, Rochester, NY 14642, USA; 2 James P Wilmot Cancer Center, University of Rochester Medical Center, Rochester, NY 14642, USA; 3 Department of Microbiology & Immunology, University of North Carolina, Chapel Hill, NC 27599, USA; 4 Department of Radiation Oncology, University of Rochester Medical Center, Rochester, NY 14642, USA

**Keywords:** angiogenesis, IL-12, VEGFR-3, image processing

## Abstract

New blood vessel formation within tumours is a critical feature for tumour growth. A major limitation in understanding this complex process has been the inability to visualise and analyse vessel formation. Here, we report on the development of a whole-tissue mount technique that allows visualisation of vessel structure. Mice expressing green fluorescent protein (GFP) made it possible to easily see GFP^+^ vessels within non-GFP-expressing B16 melanoma tumours. The small fragments of tumour used in this technique were also effectively stained with fluorescent probe-conjugated antibodies, allowing characterisation of the vessels based on surface marker phenotype. The vessels within tumour tissue were much more irregular and tortuous compared to those within surrounding normal muscle. B16 tumours stably transfected with the genes for IL-12 were used to assess the effects of this cytokine on tumour growth and vessel formation. The IL-12-expressing tumours grew more slowly and had much smaller blood vessels than the large, webbed vessels characteristic of the parental tumours, effects that were dependent on interferon gamma (IFN-*γ*). Vessels in the parental tumours were found to express VEGFR-3, the receptor for VEGF-C and VEGF-D. Expression of this receptor by the endothelial cells of the blood vessels was lost in the cytokine expressing tumours, thus suggesting a mechanism for the antiangiogenic effects of IL-12. The combination of the whole mount technique and the GFP transgenic mice provides a powerful method for visualising tumour vasculature and characterising the effects of agents such as cytokines.

Angiogenesis is a multistep process that is limited and carefully regulated in normal adult tissue, but in tumours this regulation is disrupted and the process remains ‘switched on’ (Hanahan and Folkman, 1996). Ample experimental data support the fact that tumour growth requires access to blood vessels and subsequent expansion of host vessels to provide nutrients for the growing tumour mass (Folkman, 1995a). Furthermore, many studies in a variety of tumour types have reported a correlation between the extent of tumour vasculature and poor prognosis or increased metastases (Weidner *et al*, 1991; Folkman, 1995b; Weidner and Folkman, 1996). Thus, accurate assessment of the vasculature of tumours could provide valuable information regarding treatment outcomes and the likelihood of metastatic spread to other sites. Angiogenesis can be regulated by a variety of factors. Several cytokines produced by immune cells also have been shown to affect the process of angiogenesis. One of the most noteworthy is interleukin (IL)-12, which is produced by antigen presenting cells (APC), such as macrophages and dendritic cells (DC) in response to bacterial stimuli or other inflammatory cytokines. Thus, IL-12 plays an important role in both the innate and adaptive immune responses (Trinchieri, 1998). Owing to its central role in stimulating immunity, it has been examined for possible therapeutic effects in the treatment of tumours. In addition to its effects on the immune system, IL-12 has also been shown to inhibit angiogenesis (Voest *et al*, 1995; Sgadari *et al*, 1996). Despite studies in both experimental models and in patients (reviewed in Trinchieri and Scott, 1999), and clear demonstrations of therapeutic efficacy, relatively little is known about how it alters vessel formation within tumours. In part, this is due to the difficulty in assessing the three-dimensional structure of vessels and other cellular components within the tumour. Assessment of tumour vessels is generally based on immunohistochemistry of tumour sections. Although use of this technique has led to a great deal of important information, these procedures are extremely time consuming and provide only a limited two-dimensional view of the vessels. This makes it very difficult to visualise the structure of the microvasculature and identify differences among different tumour types or changes following treatment regimens. To more easily and accurately visualise vessels within tumours, we developed a whole-tissue mount technique that provides a three-dimensional view of the tumour vasculature relative to other components of the tumour tissue. This technique was first validated by studying vessels from transgenic mice that express green fluorescent protein (GFP) (Wu *et al*, 2000), and then used to investigate the mechanism by which IL-12 influences the vessel architecture within B16 tumours.

## MATERIALS AND METHODS

### Mice

C57BL/6J and B6.129S7-*Ifng^tm1Ts^* (IFN-*γ* deficient) mice were purchased from the Jackson Laboratory (Bar Harbor, ME, USA). Green fluorescent protein-transgenic mice (C57BL/6-H-2K^b^P-GFP) were generated as previously described (Wu *et al*, 2000). The transgenic mice were bred in our animal facility at the University of Rochester, and typed prior to usage by flow cytometric analysis of peripheral lymphocytes. Guidelines for the humane treatment of animals were followed as approved by the University Committee on Animal Resources and meet the standards required by the UKCCCR guidelines (Workman *et al*, 1998).

### Construction of expression vector for IL-12

The expression vector pmIL-12-neo.1 was made by excising a 4405-bp fragment from pWRG 3169 (a kind gift from Dr Hua Yu (Rakhmilevich *et al*, 1996; Tan *et al*, 1996)), which contained the p35 and p40 subunit genes for mIL-12, each under control of a CMV promotor, using *Kpn*I and *Spe*I. This was inserted into a modified pEGFP-1 expression vector (Clontech, Palo Alto, CA, USA) in which *Kpn*I/*Xba*I sites were created by removing the EGFP gene. The final construct contained both IL-12 genes and a neo-selectable marker.

### Cell lines

The B16-F0 cell line, a C57BL/6 (H-2^b^) spontaneously arising melanoma, was obtained from the ATCC (CRL 6322) and maintained in MAT/P medium (USA patent #4.816.401) supplemented with 100 U ml^−1^ penicillin, 100 mg ml^−1^ streptomycin and 2% fetal calf serum. The B16 IL-12-expressing line (B16/IL-12) was constructed using the pmIL-12-neo.1 plasmid as described above. B16-F0 cells were transfected using Lipofectin (BRL, Gaithersburg, MD, USA), according to the manufacturer's directions. At 24 h after transfection, selection of transfectants was initiated with 400 *μ*g ml^−1^ G418 (Gibco, Grand Island, NY, USA); 10–14 days later, cells were cloned at limiting dilution. Clones were expanded and tested for IL-12 (p70) production by ELISA. This assay was specific for IL-12 p70 by virtue of the use of a rat anti-mouse IL-12 (p70) capture antibody (BD Pharmingen, clone 9A5), which reacts with the p70 heterodimer but does not react with the p40 subunit. Bound IL-12 (p70) was then detected with a biotin-conjugated anti-mouse IL-12 (p70/p40) antibody (BD Pharmingen, clone C17.8). All washes were carried out in the absence of azide, and a recombinant IL-12 (p70) standard (BD Pharmingen) at 4000 pg ml^−1^ was run in each assay.

### Tumour growth *in vivo*

Tumour cells in 100 *μ*l of Hanks balanced salt solution (HBSS) (Sigma, St Louis, MO, USA) were injected intramuscularly into the thighs of C57BL/6 or C57BL/6-H-2K^b^P-GFP mice. Mean thigh diameter was determined as described previously (McAdam *et al*, 1995). Since the B16 and B16/IL-12 tumours grew at different rates, the tumours were allowed to grow for different times and used in experiments when the thigh diameter had reached 10–13 mm.

### Immunohistochemistry and image analysis

Whole mount histology was performed by excising small pieces (∼2 mm × 3 mm × 1 mm) from the tumour and placing it on a glass slide with two drops of PBA (phosphate-buffered saline+1% bovine serum albumin+0.1% sodium azide) (Sigma, St Louis, MO, USA). A coverslip was placed on top of the piece of tumour, gently pressed down and excess PBA removed by blotting with absorbent tissue. This tumour fragment was then viewed via fluorescence microscopy. Analysis of these fragments by confocal microscopy showed them to be of fairly uniform thickness (∼100 *μ*m). To ensure uniform thickness we subsequently constructed a frame mounted to a glass slide, which placed uniform pressure on the entire perimeter of the coverslip (using screws machined to stop at 100 *μ*m) so that each fragment would be 100 *μ*m in depth. Owing to the heterogeneity inherent both within and between tumours, several pieces (six to nine) from different areas were examined from each tumour and several tumours (three to six) were used in each study.

Samples to be stained with antibodies were placed in 6 ml polypropylene tubes, and the samples blocked using Fc Block (PharMingen, San Diego, CA, USA) at 10 *μ*g ml^−1^ in 200 *μ*l of PBA with shaking at 4°C for 20 min. Primary antibody was added directly to the tube at the predetermined concentration and incubated with shaking at 4°C for 1–1.5 h. The sample was washed twice by adding 4 ml of PBA to each tube and rotating at 4°C for 1 h. For samples requiring a second step reagent, the PBA was aspirated and streptavidin-fluorochrome (PharMingen, San Diego, CA, USA) was added at 1 : 400 in 200 *μ*l PBA and incubated with shaking at 4°C for 1–1.5 h, followed by washing as described above. After the final wash, samples were removed from the tubes using 1000 *μ*l pipette tips with tip cutoff, and placed on slides as described above. To control for possible nonspecific binding of the antibodies, additional samples were stained with either a directly conjugated antibody of the same isotype as the primary antibody, or unconjugated antibody of the same isotype followed by streptavidin–biotin or streptavidin–biotin alone. In all cases, background staining was extremely low or nonexistent.

Conventional immunohistochemistry was performed using 4 *μ*m cryostat sections mounted on poly-L-lysine coated slides and stored at −80°C. Staining was performed on unfixed sections, blocked with Fc block and stained as for the whole mount technique except that 100 *μ*l of antibodies, diluted in PBA to the predetermined concentration were placed on the sections and the slides incubated in a humid box.

### Vessel quantification

The percent vessel area was determined on antibody-stained whole mounts prepared as described above. Each tumour was stained with phycoerythrin (PE)-conjugated anti-CD31 or anti-panendothelial cell (PanEC) (PharMingen, San Diego, CA, USA), and 30–35 digital images (× 10 objective) were obtained per tumour. Images were analysed using Image Pro Software by colour segmenting each image using a predefined colour range to form a binary image of the tumour blood vessels. From these images, percent vessel area was then computed for each image and averaged to obtain percent vascular area. Tumour vessel diameters were determined on whole mounts stained in the same manner. For these determinations 25–30 digital images (× 10 objective) were obtained per tumour. In each image, vessels were manually selected and diameters of all vessels were determined, using Image Pro Manual Measurement techniques, and exported to Microsoft Excel for statistical analysis. More than 500 (524–1323) vessels were measured for each tumour.

## RESULTS

### Examination of blood vessels in normal tissues of GFP mice

**Figure 1:**
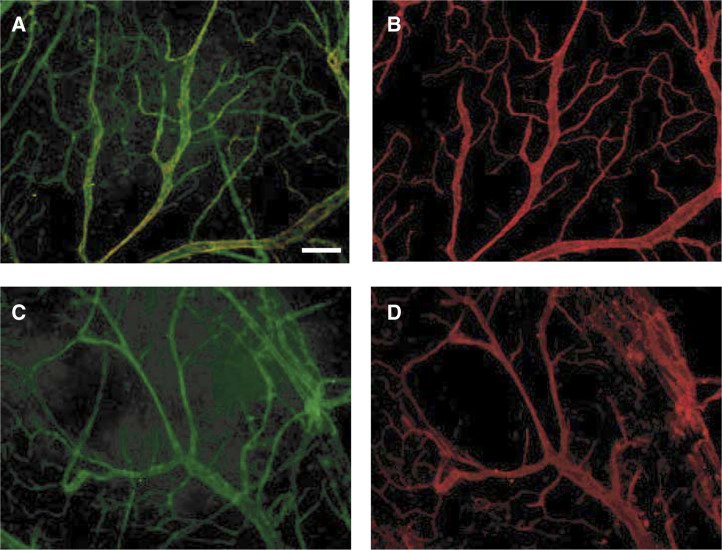
GFP expression in normal brain tissue of C57BL/6-H-2K^b^P-GFP (GFP transgenic) mice. Whole-tissue mounts of brain tissue from GFP transgenic mice were viewed using an FITC filter set (**A**, **C**) or alternatively stained with PE-anti-CD31 (**B**) or with PE-anti-H-2K^b^ (**D**) and viewed using a PE filter set. All images are at the same magnification with the white bar representing 100 *μ*m.

Expression of the GFP gene in the transgenic mice used for this study is under the control of the MHC class I promotor (Wu *et al*, 2000), and thus like class I, would be expected to be expressed in essentially all somatic tissues (Daar *et al*, 1984). To visualise GFP expression, we developed a whole-tissue mount technique, in which viable tissue was viewed microscopically essentially without manipulation (see Materials and Methods). This greatly increased the visibility of the GFP, presumably because the viable cells retained GFP expression, and also maintained much of the spatial relations within the tissue. Using this technique, we observed high levels of GFP expression in tissues such as kidney and liver (data not shown), whereas brain tissue generally expressed very low levels. These GFP expression levels correlate well with reported expression levels of class I (Daar *et al*, 1984). The brain offers a good example of a tissue that is low in both class I and GFP expression, but interestingly, it also contained structures that were strongly fluorescent, which had the morphology of blood vessels (Figure 1A,C

). To confirm the identity of these structures, we determined their expression of the adhesion molecule, PECAM-1 (platelet endothelial cell adhesion molecule) or CD31, known to be strongly expressed on endothelial cells lining blood vessels (Figure 1B). We speculated that sufficient antibodies might be able to penetrate the small tumour pieces. The tumour fragments were incubated with PE-conjugated anti-CD31, and the antibody was found to penetrate readily the brain tissue and stain the GFP^+^ structures, thus confirming that they were indeed blood vessels. Fragments of the brain tissue were incubated with a PE-conjugated anti-class I antibody, which also stained the blood vessels quite brightly, whereas the surrounding brain tissue was largely unstained (Figure 1D). Fragments stained with a PE-isotype control showed minimal staining (data not shown). Thus, the increased class I expression on the vessels provided an explanation for the observed greater expression of GFP. These results with normal brain tissue indicated that blood vessels should be visible within implanted GFP-negative tumour tissue, and confirmed that fluorochrome-conjugated antibodies could penetrate the tissues.

### Blood vessels in B16 tumours (GFP and CD31 staining)

**Figure 2:**
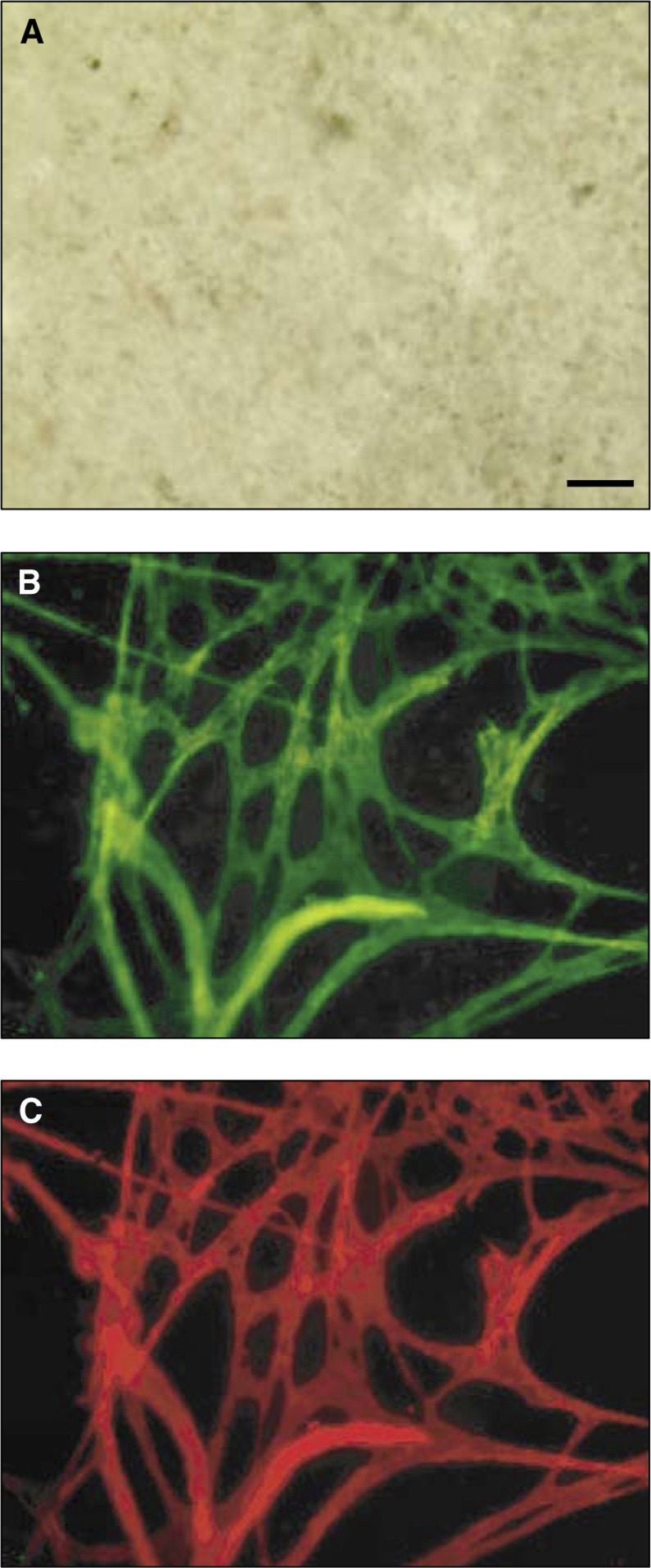
GFP^+^ blood vessels in parental B16 tumours. A whole-tissue mount preparation of a parental B16 tumour grown in a GFP^+^ mouse, stained with PE-anti-CD31 and viewed by (**A**) bright-field or (**B**) fluorescence using a GFP filter set or a (**C**) PE-filter set. The images are all of the same field with the bar representing 100 *μ*m.

To assess vessel formation within growing tumours, B16 melanoma cells were injected intramuscularly into the hindlimbs of GFP transgenic mice and allowed to grow until the leg diameter was 10–13 mm at which time the tumours were removed. Since B16 tumours grow as soft masses of tissue, it was very easy to prepare the whole mounts, which allowed visualisation of the tumour vessels with apparently minimal disturbance of the spatial relations. Although the tissue viewed by bright field was unremarkable (Figure 2A

), the same tissue viewed for green fluorescence revealed a rich network of blood vessels visible as complete three-dimensional structures (Figure 2B). The vascular identity of these structures was verified by staining with PE-anti-CD31 (Figure 2C). As with the brain tissue, the antibodies were found to penetrate throughout the small fragments of tumour tissue, and samples stained with a PE-isotype control showed negligible staining (data not shown).

### Growth kinetics of parental and cytokine-expressing tumours

**Figure 3:**
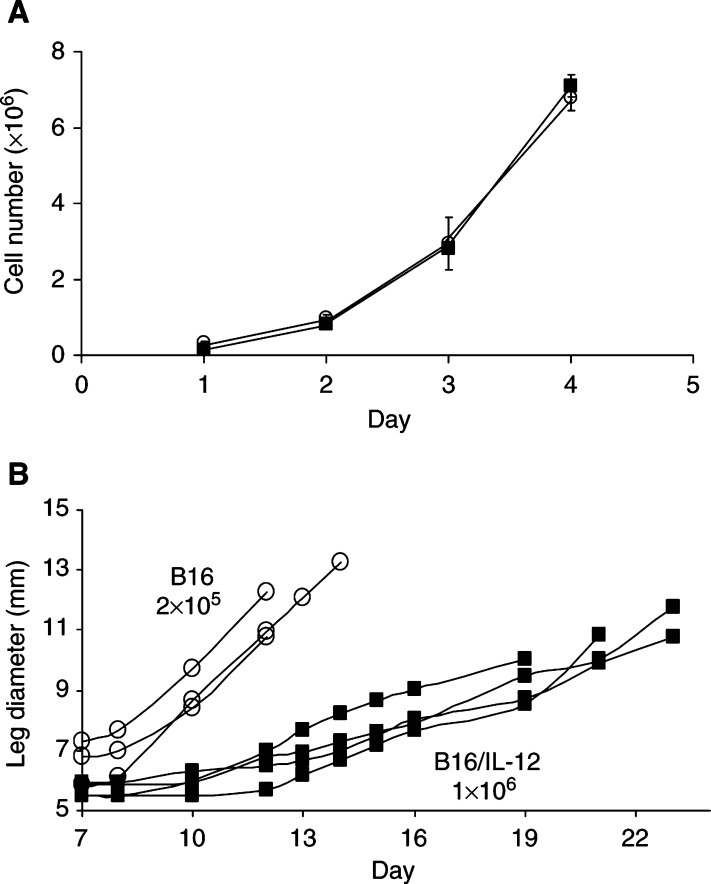
Growth of B16 parental and cytokine-transfected tumour cells *in vitro* and *in vivo* in GFP mice. (**A**) 1 × 10^5^ parental (circles) or IL-12-transfected (squares) B16 tumour cells were plated *in vitro*. At daily intervals, cells were removed from triplicate plates and counted individually. Points are the mean and standard deviation of the counts. (**B**) 2 × 10^5^ parental (circles), or 1 × 10^6^ B16/IL-12 (squares) tumour cells were implanted intramuscularly in the leg of syngeneic C57BL/6-H-2K^b^P-GFP mice and tumour growth followed by measuring the leg diameter. Each line represents tumour growth in an individual mouse.

Previous studies have shown that local production of cytokines within tumours can have a marked effect on vascularisation. In particular, IL-12 has been shown to inhibit angiogenesis (Voest *et al*, 1995). However, little information is available on the three-dimensional structure of the vessels or the mechanism of action of IL-12. In the current study, we have used the whole mount technique to examine IL-12-expressing tumours and to address these issues. B16 tumours were transfected with the gene for IL-12 as previously described and stable transfectants selected. The stably transfected clone used in these studies produced an average of 10 ng IL-12 per 10^6^ cells and proliferated at the same rate *in vitro* as the untransfected cells (Figure 3A

). Preliminary experiments revealed that the IL-12-expressing tumours implanted in C57BL/6 mice grew much slower than the parental or mock transfected tumours (data not shown). To match the parental B16 tumour growth rates more closely, the number of transfected cells injected was increased five-fold to 1 × 10^6^ cells. Mice injected with this number of cells had tumour bearing leg diameters of approximately 12 mm by 20–24 days post-injection. Figure 3B shows representative growth curves for these tumours. Even with injection of an increased number of cells, the IL-12-expressing tumours grew more slowly than the parental tumours.

### Analysis of vessel morphology in parental and IL-12-expressing tumours

**Figure 4:**
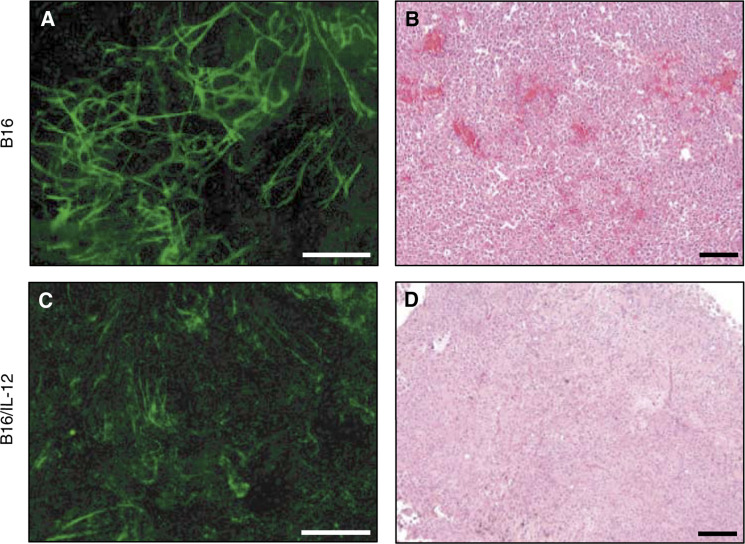
Varying GFP fluorescence intensity of blood vessels in parental and cytokine-expressing tumours. Montages (16 microscopic fields) of whole-tissue mounts of (**A**) B16 and (**C**) B16/IL-12 were prepared and imaged under identical conditions. Each montage illustrates a 3.2 mm × 4.2 mm tissue piece. The white bar represents 750 *μ*m. Paraffin sections from (**B**) B16 or (**D**) B16/IL-12 tumours were stained with haematoxylin and eosin. The black bars in (B) and (D) represent 100 *μ*m.

Whole mount preparations for these tumours revealed striking differences in the vasculature between the parental and IL-12-expressing B16 tumours that may explain the altered tumour growth observed. Images composed of montages of 16 microscope fields (× 10 objective) encompassing an area of approximately 3.2 × 4.2 mm were prepared from pieces of size matched B16 and B16/IL-12 tumours (Figure 4A,C

). Vessels in the parental tumours appear larger and are much easier to discern than are the vessels in the IL-12-expressing tumours. Paraffin sections of each type of tumour were also prepared and stained with haematoxylin and eosin to visualise the morphology of these tumours by conventional histology (Figure 4B,D). The sections from both the parental and IL-12-expressing tumours show regions of viable tissue taken from progressively growing tumours. The parental tumour tissue appears to have more vessels as well as ‘blood lakes’, regions of pooled blood resulting from leaky and haemorrhagic vessels often observed within tumours (Yancopoulos *et al*, 2000).

**Figure 5:**
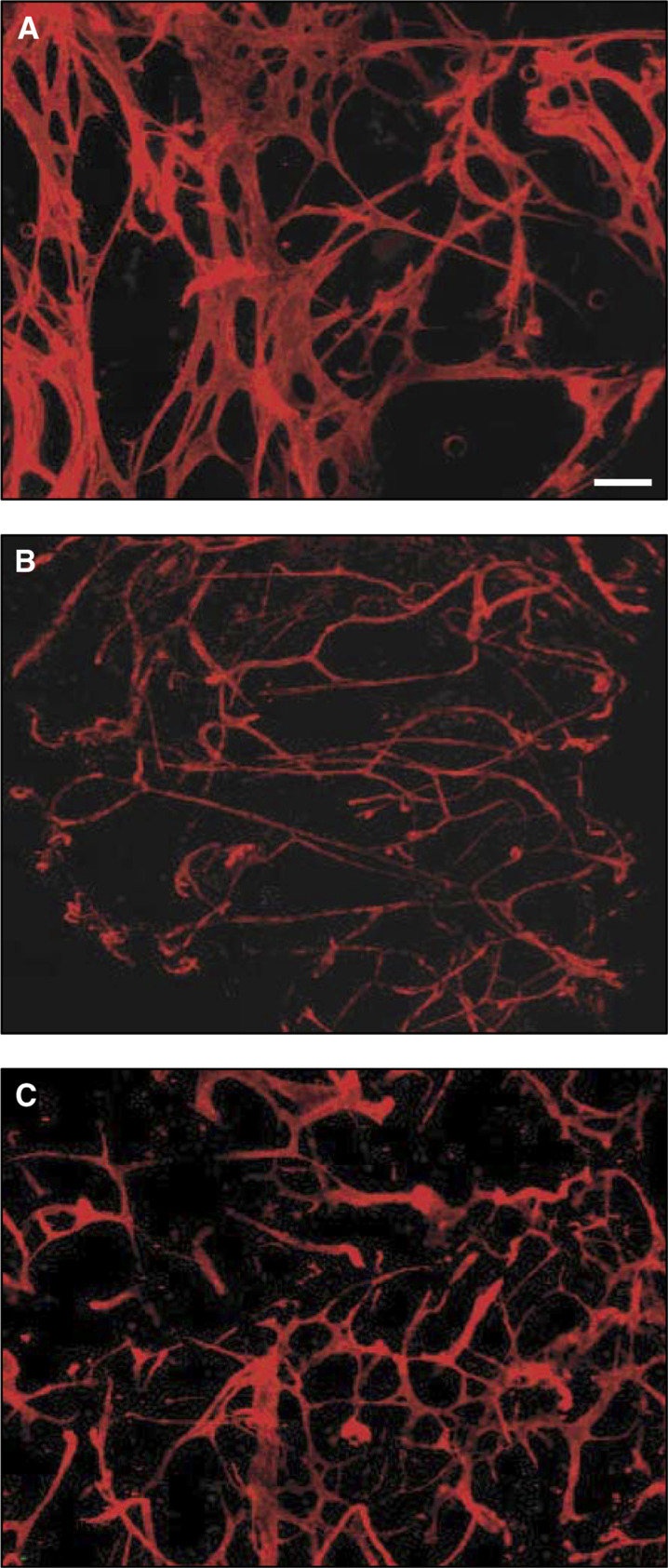
Visualisation of altered blood vessel morphology in cytokine-expressing tumours. Whole-tumour mounts were prepared from (**A**) B16, or (**B**) B16/IL-12 tumours grown in normal mice or (**C**) B16/IL-12 tumours grown in IFN-*γ*-deficient mice and stained with biotin-conjugated anti-panEC and PE–streptavidin. All photographs are of the same magnification with the white bar representing 200 *μ*m.

To distinguish more easily the morphology differences and to visualise the vessels exclusively, the tumour fragments were stained with biotin-conjugated MECA-32, an antibody to the panEC antigen and detected with PE–streptavidin (Figure 5

). This antigen is expressed on endothelial cells, but not leucocytes (Leppink *et al*, 1989; Hallmann *et al*, 1995), thus making it possible to observe the vessels easily without visualising the GFP^+^ infiltrated host cells. The large, webbed or honeycombed vessels observed in the parental B16 tumours are characteristic of immature angiogenic vessels that have not yet undergone extensive remodelling. The whole mount technique provides an excellent three-dimensional view of these newly formed vessels. In contrast, the vessels in the IL-12-expressing tumours had a much different morphology. They appeared to be much smaller in diameter, and also longer and less convoluted than the vessels in the parental tumours.

### Role of IFN-*γ* in the IL-12-mediated tumour effects

To better understand the mechanism by which IL-12 exerts its growth inhibitory and vascular effects in the B16-tumour system, we examined the role of downstream cytokines, particularly IFN-*γ*. To assess the role of IFN-*γ* in the vessel changes observed in the B16/IL-12 tumours, we also implanted the IL-12-expressing tumour cells into IFN-*γ*-deficient mice. As can be seen from the image in Figure 5C, the vessels in the B16/IL-12 tumours growing in these mice are intermediate in size and morphology compared to those in the parental and the IL-12 tumours growing in wild-type mice. The tumours also grew faster in these mice compared to the same cells in C57BL/6 mice, with kinetics similar to parental cells (data not shown). This intermediate vascular phenotype and reversal of the tumour growth inhibition indicate that IFN-*γ* is involved in the IL-12-mediated effects in this system, although it does not account for all of the effects.

**Figure 6:**
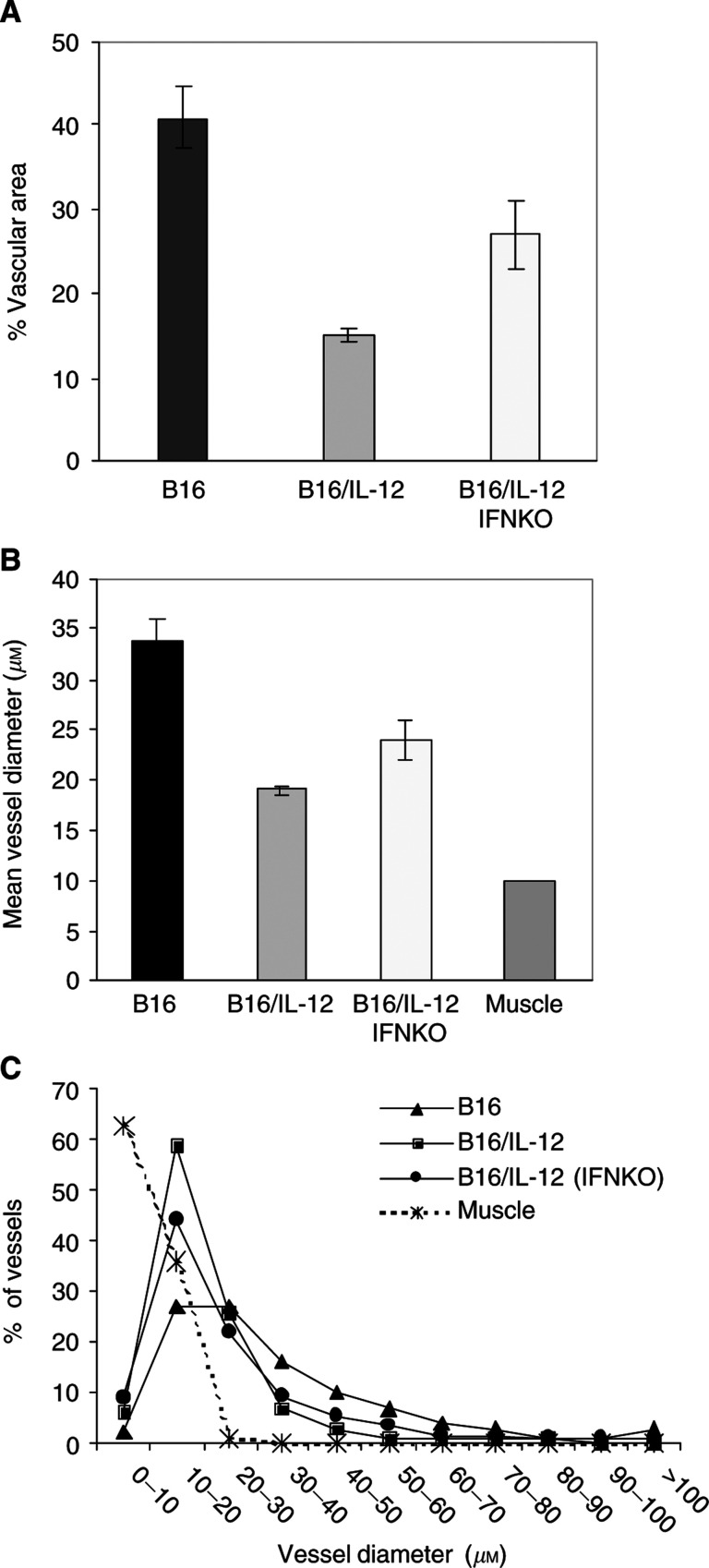
Quantification of blood vessel diameter and area in parental and cytokine-expressing tumours. (**A**) Percent vessel area was determined for each of the three tumour types using whole mounts stained for either CD31 or PanEC on 30–35 images (× 10 objective) per tumour and analysed as detailed in Materials and Methods. Three B16, four B16/IL-12 in C57BL/6 mice and two B16/IL-12 tumours in the IFN-*γ*-deficient mice were analysed. Significance as determined by the Bonferroni test, was *P*<0.001 for B16 compared to B16/IL-12 in wild-type mice and *P*=0.012 for B16 compared to B16/IL-12 in IFN-*γ*-deficient mice. There was no significant difference between B16/IL-12 grown in wild-type and deficient mice. (**B**) Average vessel diameter was determined using Image Pro software on whole mounts stained for CD31, and based on 25–30 images (× 10 objective) for each tumour. Data shown are mean and standard error of 524–1323 vessel diameters per tumour type. For comparison, vessels from normal leg muscle were also analysed. Significance was *P*<0.001 for B16 compared to B16/IL-12 in wild-type mice and *P*=0.009 for B16 compared to B16/IL-12 in IFN-*γ*-deficient mice. There was no significant difference between B16/IL-12 grown in wild-type and deficient mice. (**C**) Vessel diameter distribution, based on 10 *μ*m intervals.

The alterations in vasculature between the parental and the IL-12-expressing tumours were quantified by measuring two parameters. The first parameter involved determining the percentage of the field that was occupied by vessels, which provides the relative amount of vascular area within each tumour type. Using this measure, the IL-12-expressing tumours had significantly less vascular area than did the parental tumours (Figure 6A

), whereas the vascular area of the IL-12-expressing tumours grown in the IFN-*γ*-deficient mice was intermediate. The second parameter measured was the diameter of the vessels present in each tumour type, which was determined by measuring all vessels in several fields. For these measurements, an individual vessel was defined as the unbranched segment between junctions. Mean vessel diameters in the IL-12-expressing tumours were significantly smaller than in the parental tumours when both were grown in wild-type mice. As suggested by the images of Figure 5, the vessels in the IL-12-expressing tumours grown in the IFN-*γ*-deficient animals were intermediate in diameter. By this measure, the vessels in the IL-12-expressing tumours grown in the IFN-*γ*-deficient animals were also significantly different from the parental cells, but not different from the IL-12-expressing tumours grown in wild-type mice. In all three cases, the tumour vessels had a significantly larger diameter than vessels from normal muscle. The data are also shown as a frequency histogram (Figure 6C), which clearly shows that the differences are primarily in vessels with diameters from 10 to 30 *μ*m.

### Altered expression of vascular endothelial growth factor (VEGF) receptor 3 on blood vessels in IL-12-expressing tumours

**Figure 7:**
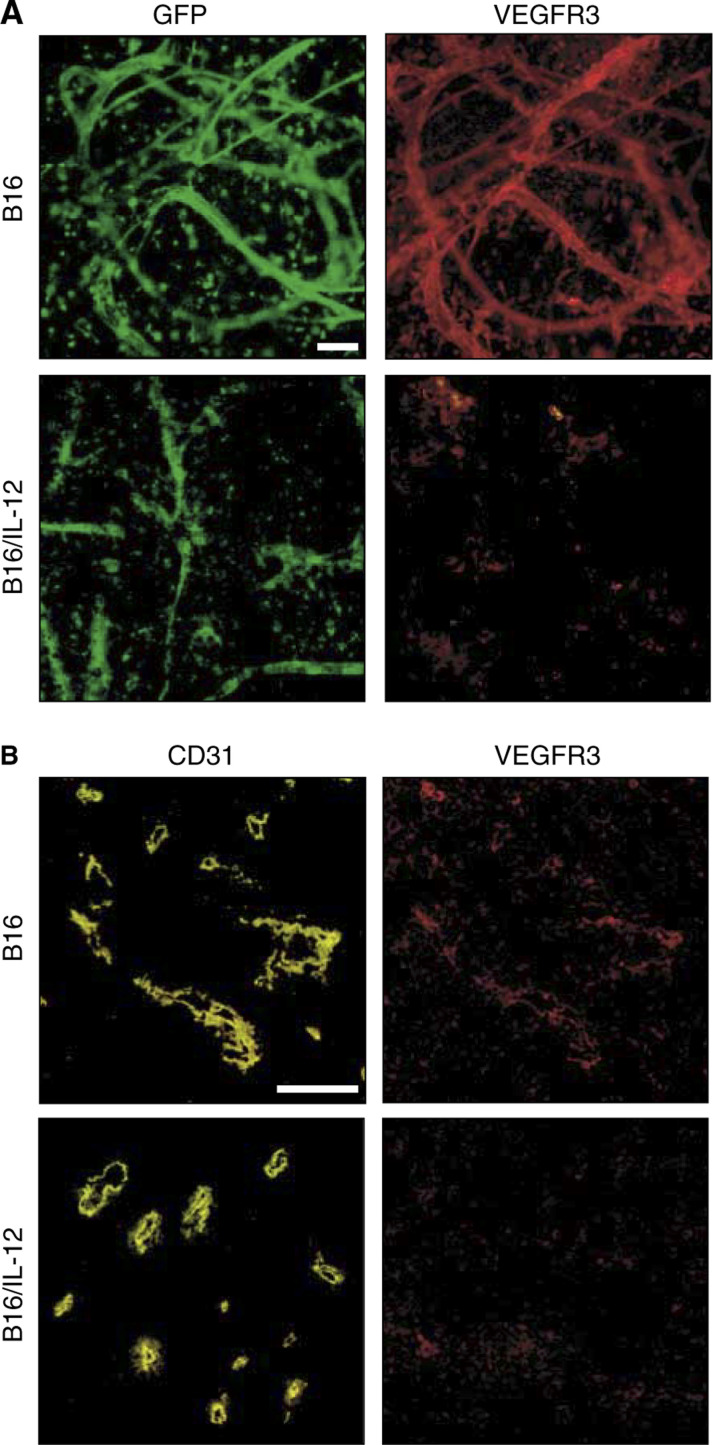
Altered expression of VEGFR-3 on blood vessels in IL-12-expressing tumours. (**A**) Whole mount preparations of B16 (top row) and B16/IL-12 (bottom row) tumours were stained with biotin-conjugated anti-VEGFR-3 and PE–streptavidin and viewed for green fluorescence (left images) or red fluorescence (right images). All images are of the same magnification; the bar represents 100 *μ*m. (**B**) Cryrostat sections (4 *μ*m) were prepared from B16 (top row) and B16/IL-12 (bottom row) tumours and stained with PE-anti-CD31 and with biotin-conjugated anti-VEGFR-3 and APC–streptavidin and viewed for PE fluorescence (left images) or APC fluorescence (right images). All images are of the same magnification; the bar represents 100 *μ*m.

To better understand the mechanism of action of the IL-12-mediated changes in vessel structure and morphology, we analysed one of the most potent angiogenic factors expressed by tumour cells, vascular endothelial growth factor (VEGF) and its corresponding receptors (VEGFR). Antibodies to VEGFR-1, VEGFR-2 and VEGFR-3 were used to stain vessels from the parental and IL-12-expressing tumours. Both VEGFR-1 and VEGFR-2 were found on vessels from both types of tumours and thus were unlikely to account for the differences in vasculature between the two types of tumour (data not shown). In contrast, VEGFR-3 expression, which was clearly present on the vessels from the parental tumours, was highly downregulated on the vessels from the IL-12-expressing tumours. This can be clearly seen in the images in Figure 7A

in which the parental tumour GFP^+^ blood vessels also stain with the PE-anti-VEGFR-3 (top row of images). In the B16/IL-12 tumours, GFP^+^ blood vessels were also clearly visible, but these were completely unstained with the anti-VEGFR-3 antibody (bottom row of images), even though other cell types are seen to be VEGFR-3 positive. To verify this finding, additional tumours were examined by conventional immunohistochemistry (Figure 7B). For these studies, blood vessels were identified by staining with anti-CD31-PE and the biotin-conjugated VEGFR-3 antibody was detected with streptavidin–APC. As with the whole mount technique, immunohistochemistry of sections revealed VEGFR-3 expressed on vessels in the parental tumours but not on vessels in the IL-12-expressing tumours. Thus, IL-12 appears to limit the upregulation of VEGFR-3 on the vessels, thus preventing stimulation of their growth by its known ligands, VEGF-C and VEGF-D.

## DISCUSSION

This study describes and validates a new whole mount method for visualising and characterising the blood vessels within experimental mouse tumours. The use of transgenic mice expressing GFP under the control of the class I promotor greatly assisted verification of the technique. In initial studies, we attempted to use conventional immunocytochemistry to visualise blood vessels, but encountered problems in detection of the GFP fluorescence (data not shown), as has been previously described (Sullivan and Kay, 1999). Several factors probably contribute to this diminution of the fluorescence. First, very thin sections generally are used (10 *μ*m or less) and since many of the vessels are cut as cross-sections, this means that only two to three cells are being observed, resulting in limited fluorescence intensity. Even with frozen sections, it is typical to use some fixation method, which tends to affect the GFP fluorescence intensity adversely. In contrast, the whole mount method uses tissue fragments, making it possible to view large segments (and hundreds of endothelial cells) of essentially intact vessels. The tissue is neither fixed nor frozen, so GFP expression is maintained at a high level within the viable cells. Compared to conventional immunohistochemistry, this method is simpler, faster and provides a more comprehensive view of the vascular configuration within the tumour tissue.

Since the implanted tumours are GFP negative, they provide a blank background on which the bright GFP fluorescent blood vessels of host origin are easily seen. To further characterise the blood vessels, we used fluorochrome-conjugated antibodies specific for surface markers present on the endothelial cells. Despite the relatively large size of these antibody molecules, they were found to diffuse throughout the tissue. All of the antibodies used in these experiments were of the IgG class, which is known to be present in tissues and may have been selected over evolutionary time to be able to penetrate many tissues and organs easily. Early experiments established the effectiveness of the staining by verifying that PE-anti-CD31-stained vessels and GFP^+^ vessels were colocalized. The ability to stain with antibodies greatly expands the power of the whole mount technique in that it enables one to use the large number of fluorochrome-conjugated reagents that have been developed for flow cytometry. Thus, this technique combines many of the best features of flow cytometry and immunohistochemistry in a method that is simple to perform.

We have used this technique to determine how IL-12 alters the angiogenic properties of tumour cells. Our results indicate that IL-12 inhibits angiogenesis in B16 mouse tumours by downregulating the expression of VEGFR-3. By using B16 tumour cells transfected with the genes for IL-12, we were able to grow tumours in which the cytokine was produced and secreted locally within the tumour microenvironment. This ensured high local concentrations of the cytokine while limiting side effects evident when this cytokine is delivered systemically (Car *et al*, 1999). This local production of the cytokine greatly decreased the tumorigenicity of the tumour cells, consistent with earlier reports for B16 tumours treated systemically (Brunda *et al*, 1993; Smyth *et al*, 2000) and engineered to produce IL-12 locally (Nanni *et al*, 1998). These previous studies (Coughlin *et al*, 1995, 1997, 1998; Sgadari *et al*, 1996) focused mainly on the role of IL-12 in stimulating immune cells to alter tumorigenicity, but other studies have also examined IL-12's effects on angiogenesis in additional tumour model systems when the cytokine was delivered systemically. IL-12 has been shown to regulate the level of VEGF-A in a mouse mammary tumour model (Dias *et al*, 1998). However, the marked differences in the morphology of the blood vessels in the IL-12-expressing tumours have not been described previously, and there is no prior information available on the mechanisms involved in the alteration of vessel formation by these cytokines.

There is compelling evidence that angiogenesis in tumours is initiated by the hypoxic conditions that develop as a result of oxygen consumption by rapidly growing tumour cells (Vaupel, 1977; Pilch *et al*, 2001). The hypoxia stimulates the tumour cells to increase production of various angiogenic factors, of which the VEGF family members and their receptors play a dominant role in many tumour models (Veikkola and Alitalo, 1999). The primary member is VEGF-A, which is expressed by many tumour cells and binds to VEGFR-2, thought to be the main signal transducing receptor (Veikkola *et al*, 2000). Recently, two other family members, VEGF-C and VEGF-D, have also been implicated in tumour angiogenesis (Cao *et al*, 1998; Valtola *et al*, 1999; White *et al*, 2002). These ligands, like VEGF-A, bind VEGFR-2 but in addition also bind VEGFR-3 (Taipale *et al*, 1999). We have shown that mRNA for VEGF-A, -B, -C and -D is present within B16 and B16/IL-12 tumours grown *in vivo* (data not shown). Initially, it was thought that VEGFR-2 was expressed on both blood vascular and lymphatic endothelial cells, whereas VEGFR-3 expression was limited to lymphatic vessels and fenestrated endothelium (Partanen *et al*, 2000). However, more recently it has been clearly demonstrated that VEGFR-3 expression is upregulated on the blood vessels within tumours of multiple types including melanoma, colon and breast (Valtola *et al*, 1999; Achen *et al*, 2001; White *et al*, 2002). A recent paper has suggested that expression of VEGFR-3 could be used as a progression marker in human cutaneous melanoma (Clarijs *et al*, 2002). In the B16 mouse model used in these studies, expression of both VEGFR-2 and -3 was detected on the blood vessels within the tumours, and angiogenesis within the tumours was clearly evident. However, in the tumours expressing IL-12, VEGFR-2 was present, but the vessels failed to upregulate VEGFR-3. The vessels within these IL-12-expressing tumours were small and less numerous, with little evidence of any active angiogenic process. It would thus appear that prevention of VEGFR-3 expression is an important mechanism in the antiangiogenic effects of IL-12. Furthermore, this result suggests that the ligands for VEGFR-3, VEGF-C and VEGF-D may play an important role in the angiogenesis observed in this tumour system. It is interesting that although these ligands, like VEGF-A, can also bind to VEGFR-2, the downregulation of VEGFR-3 by IL-12 still has a marked effect on the tumour vasculature despite the unaltered expression of VEGFR-2 in the presence of IL-12. At least for the B16-tumour system, VEGFR-3 appears to play a greater role in tumour angiogenesis than previously appreciated.

The IL-12-mediated effects we observed in this model tumour system appear to be mediated primarily via IFN-*γ*. Injection of the IL-12-transduced tumour cells into IFN-*γ*-deficient mice resulted in tumours that grew with kinetics similar to the parental tumours (data not shown), and the vessel morphology was similar (although not identical) to that of the vessels in the parental tumour and the vessels expressed the VEGFR-3 (data not shown). In some tumour models, the IL-12 effects have been shown to require tumour cells that are responsive to IFN-*γ* (Coughlin *et al*, 1998), implying that the tumour cells are producing additional factors in response to IFN-*γ* that subsequently alter the angiogenic process. Candidate molecules for this activity include the chemokines, IP-10 and MIP-1, although it is not completely clear how these molecules function to suppress angiogenesis. It will be interesting to determine whether the shared receptor for these molecules, CXCR3, which is expressed on activated T cells is also expressed on the vascular endothelial cells within the tumours, as has recently been shown for human kidney tumours (Romagnani *et al*, 2001). An earlier *in vitro* study has indicated that IL-12-activated CD4^+^ and CD8^+^ T cells, as well as natural killer (NK) cells can affect the function of endothelial cells (Strasly *et al*, 2001). These cells may also play a role in regulation by IL-12 of VEGFR-3 expression on endothelial cells within tumours.

By using whole mounts of viable tumour tissue growing in GFP^+^ transgenic mice, we have been able to visualise the vasculature within B16 tumours and observe the effects of locally produced IL-12 on the tumour vessels. This technique combined with immunohistochemistry has allowed us to determine that the antiangiogenic effect of IL-12, at least in this tumour model is related to the marked downregulation of VEGFR-3 expression on the endothelial cells of the tumour vasculature. These results emphasise the importance of these growth factor receptors in the angiogenic process and suggest new possibilities for tumour control.
